# Experimental Evaluation of Dose-Dependent Hepatorenal Toxicity of Traditionally Prepared Arak in Swiss Albino Mice

**DOI:** 10.1155/jnme/9304159

**Published:** 2025-06-16

**Authors:** Rebuma Sorsa, Niguse Hamba, Daba Abdissa, Zelalem Banjaw, Hawi Gobena, Muntaha Hamza, Melese Abere, Tilahun Alemayehu Nigatu

**Affiliations:** ^1^Department of Biomedical Sciences, Jimma University, Jimma, Ethiopia; ^2^Department of Nursing, Jimma University, Jimma, Ethiopia; ^3^Department of Biology, Jimma University, Jimma, Ethiopia; ^4^Department of Pathology, Jimma University, Jimma, Ethiopia

**Keywords:** Arak, hepatotoxicity, histopathology, inflammation, necrosis, nephrotoxicity

## Abstract

**Background:** Arak is the most popular traditional alcohol in Ethiopia. Although it is widely consumed across the country, its effects on different organs have not been well studied yet. Thus, this study aims to evaluate the dose-dependent hepatorenal toxicity of traditionally prepared Arak in Swiss albino mice.

**Methods:** The investigation utilized 24 newly bred Swiss albino mice (12 males and 12 females) aged 8–10 weeks, divided into four groups of six individuals each (three males and three females), with Group I receiving 1 mL of distilled water/kg, and Groups II–IV receiving 1 mL/kg of 20%, 40%, and 45% Arak, respectively, orally on daily basis for six weeks; the investigation included blood, urea and nitrogen; creatinine; aspartate transaminase; alanine aminotransferase; and histological examination.

**Results:** The study found that Arak and its metabolite, ethanol, have a dose-dependent negative impact on the liver and kidney's microstructures, and Arak has a significant dose-dependent effect on decreasing body weight, increasing serum levels of aspartate transaminase and alanine aminotransferase, and elevating serum levels of blood, urea, and nitrogen and creatinine in Swiss albino mice. Higher doses of 1 mL of 40% and 1 mL of 45% Arak/kg caused inflammation, edema, obscured Bowman's capsule, foamy appearance, and necrosis, while lower doses of 1 mL of 20% Arak/kg had a lesser impact. Further research is needed to evaluate the effect of Arak on human hepatorenal structures and biomarkers.

## 1. Introduction

Ethiopia is home to a wide range of traditional alcoholic beverages that hold significant communal and cultural value [1, 2]. Arak is the most popular homemade alcoholic drink, deeply embedded in the social fabric of rural and semiurban communities in particular [3, 4]. The distillation process of the fermented mixture takes around 40 min using traditional firewood methods [1]. Even though Arak's alcoholic content varies based on the raw materials and production method used, it mostly ranges between 30% and 50% (vol/vol of ethanol), with a pH of around 4. It is often consumed during holidays, recreation, church festivals, and funerals as a stimulant believed to facilitate interpersonal relationships and is part of human dietary culture [3–9].

In 2016, alcohol consumption resulted in 3 million deaths and 132.6 million DALYs, which was reported by approximately 2.3 billion people globally [9, 10]. Even though the Ethiopian government has implemented an alcohol control policy, consumption trends continue to move unfavorably [11–15]. This unfavorable consumption leads to different nutritional and metabolic disorders [16, 17]. With prolonged Arak consumption, permanent damage can occur [18].

Alcohol can harm the organs by reducing function, changing microarchitecture, and causing diuresis, especially as Arak may contain varying concentrations of alcohol, which may influence its toxicity [19, 20]. It can induce the microsomal ethanol oxidation system and lead to reactive oxygen species [21]. Skeletal muscle damage and oxidative stress can cause renal tubular injury and hepatorenal syndrome [22, 23]. Elevated urea and creatinine levels in alcoholics may occur in advanced disease but are not always indicative of kidney injury [24, 25].

Despite the cultural significance and prevalence of consumption of Arak in different societies, there is a lack of comprehensive research evaluating its dose-dependent hepatorenal toxicity. This study aims to evaluate the dose-dependent hepatorenal toxicity of traditionally prepared Arak in Swiss albino mice. The findings from this study inform consumers about their health and raise awareness of the potential health risks associated with Arak.

## 2. Materials and Methods

### 2.1. Experimental Animal Preparations and Handling

The study utilized 24 newly bred adult Swiss albino mice (12 males and 12 females). This sample size was determined by the resource equation approach, which considers degrees of freedom “E” between 10 and 20 (at least five animals in each group for cross-tabulation) to balance the constrained resources [26]. The mice weighing between 30 and 42 g and aged 8–10 weeks were obtained from the JUCAVM animal experimentation and biotechnology laboratory. The mice were labeled and randomly allocated into four groups (three males and three females in each group). Those grouped mice were housed separately in stainless-steel and aluminum cages, bedded with clean husk. The cages were cleaned twice per week, and the husk was changed daily to ensure experimental animal welfare standards [27]. The mice were maintained in a room with a temperature of 20°C–26°C and a 12 h light/12 h dark cycle, provided with a standard free rat diet and clean tap water except during starvation at the end of the experiment. The food changed every 12 h, and residual chow was discarded; before the study, the mice were acclimatized to laboratory conditions for 1 week to minimize environmental-specific stress, as recommended in similar studies [28–31].

### 2.2. Arak Sample Collection and Preparation

Before the experiment, Arak was prepared for this study purpose by the researcher since it is difficult to characterize the ingredients of Arak prepared by other producers/sellers. Then, Arak was collected directly from three randomly selected producers' clean glass containers in Bacho woreda, Tulu Bolo town in the southwest Shoa zone, to compare the toxicity level during experimentation. The alcoholic contents of the Arak were measured using an alcoholometer, while its pH was determined using a pH meter at the Chemical Engineering Laboratory of the Jimma Institute of Technology.

### 2.3. Experimental Design

The study involved four groups of six mice each (3 males and 3 females) receiving different doses of Arak, with Group I receiving distilled water and Groups II–IV receiving 1 mL/kg of 20%, 40%, and 45% Arak, respectively, daily for six weeks. The doses were determined according to a previous study by Hassan et al. [32]. The mice were acclimatized to the laboratory environment for two weeks before experimentation, with each experimental unit consisting of three male and four female mice in a separate cage; Arak was provided to each mouse orally in the experimental group once daily after measuring the dose using an insulin syringe, while the placebo group received distilled water. Each mouse was coded, and the dose was registered to avoid duplication of the experiment; after experimentation, the mice were monitored for any behavioral changes for two hours.

### 2.4. Euthanasia and Organ Sample Collections

After completion of the experiment, the mice underwent overnight starvation of food (but not water) to prevent postoperative nausea and vomiting, followed by anesthesia, using the equipment recommended by Edinaldo et al. (2011), which included an air compressor, container for diethyl ether or inhalation agent, inhalation chamber, three-way stopcock for control of the anesthetic agent, and anesthetic mask, with diethyl ether 5% inhalational anesthesia used to maintain anesthesia during the surgical procedure, administered by a veterinary or animal science expert [32]. After anesthetizing, the mice blood sample was collected for biochemical analysis. Motor movement and corneal reflex were checked, and immobility was observed using a magnification mirror to ensure effective anesthesia before making any incisions in the midline abdominal area to remove the liver and kidney of the mice. Then, the mouse was sacrificed by an overdose of anesthesia, which is the ideal mode of euthanasia with minimum pain, suffering, or distress.

### 2.5. Processing of Organ Samples for Histopathological Study

After 42 days, hepatic tissue portions were taken via a sterile surgical blade from the neck-to-pubic incision and preserved in a 10% neutral buffered solution in a labeled transparent glass container for gross and histopathological examination by a pathologist. The tissue was fixed, washed with water, dehydrated with ascending alcohol concentrations, cleaned with xylene, and infiltrated in three changes of paraffin wax for histopathological procedures.

Tissue blocks were formed by inserting tissues into paraffin wax on plastic plates, labeled, and preserved at room temperature until sectioned. The tissue blocks were cut into 5 mm-thick sections using a ribbon microtome and placed on precleaned slides, stretched out with egg albumin, fixed to the slides in an oven, stained with hematoxylin and eosin, and prepared for regular H and E staining. To remove paraffin, the tissue was placed in Xylene I and II, hydrated with descending concentrations of alcohol, and prepared for histological examination. Tissue sections were washed with tap water, stained with hematoxylin for 6 min, submerged in acidic alcohol for distinction, and counterstained with eosin for 15 s. The sections were then washed with tap water for two minutes, blued in a blueing solution, and dehydrated with increasing alcohol concentrations of 50%, 70%, and 95% for two minutes [33].

The dehydrated parts were cleared with Xylene I and Xylene II for three minutes each, permanently placed on microscopic slides using DPX and cover slips, and observed under a light microscope by a histologist and pathologist to investigate any histological changes, contrasting the consumed groups with the control group. Photomicrographs of selected slides of liver and kidney from both the consumed and control mice were taken under a light microscope with 40X magnification, and photomicrographs were taken objectively using an automated built-in digital photo camera at the histology lab of Jimma University.

### 2.6. Data Quality

During the study period, an experienced laboratory technician and principal investigator handled the animals and provided Arak according to the protocol. Histological techniques and principles were strictly followed during tissue collection, preservation, processing, and slide preparation. Blinding was applied during administration and outcome assessment by senior histologists and pathologists to the groups and doses to eliminate observer bias during microscopic slide examination. All prepared slides were re-examined to ensure the consistency of the findings.

### 2.7. Body Weight and Hepatorenal Biomarkers

The liver damage biomarkers, AST and ALT, as well as the renal damage biomarkers, BUN and creatinine, were measured according to standard principles and procedures outlined in the manufacturer's manual for the diagnostic kits.

### 2.8. Blood Collection and Serum Preparation

Six weeks after the experiment began, a cardiac puncture was performed, and approximately 2–2.5 mL of blood sample was collected from each mouse and placed in a serum-separating test tube. The blood samples were then left to clot at room temperature for 30 min. After centrifugation at 3000 rpm for 10 min, the serum was separated using a micropipette (1000 μL) and stored at -20°C until it was analyzed for liver and kidney biomarkers. Then, the mice were euthanized by cervical dislocation following anesthesia.

### 2.9. Statistical Analysis

The raw data from the animals in experimental groups (i.e., weight, AST, ALT, BUN, and creatinine) were cleaned, coded, and analyzed using SPSS Version 25. For quantitative data, continuous variables (weight, AST, ALT, BUN, and creatinine level) of each group were analyzed using one-way ANOVA and post hoc (Tukey) to determine significant differences between the groups (*p* value < 0.05). For qualitative data, microscopic slides were prepared for histopathological examination of kidney and liver tissue. The evaluation of liver and kidney tissue features was performed by a senior pathologist and histologist for any pathological alteration and normal appearance, respectively.

### 2.10. Ethical Approval

The study was approved by the Institutional Review Board of Jimma University, or the Institute of Health Research Postgraduate Directorate (Ref. no. IHRPGD835/2020), and COVID-19 prevention measures were implemented during the research. The experimentation procedures involving mice followed the Ethiopian Public Health Institute's animal handling and treatment guidelines and were reviewed by veterinary doctors to ensure they were appropriate and humane. The adverse effects of Arak were prevented by providing it after feeding and adjusting the dose based on mouse weight. Unnecessary duplication of the experiment was avoided by following the protocol. The mice were handled to minimize distress and pain, and anesthesia was provided and checked for pain sensation before dissection or euthanasia. Personal protective equipment was worn during procedures on Swiss albino mice, and any remnant body parts were discarded or buried in sealed containers to prevent environmental contamination, following the Universal Declaration of Animal Welfare [16, 19].

## 3. Results

### 3.1. Concentration of Arak Collected

The concentrations of ethanol in the randomly prepared Arak were found to be 20%, 40%, and 45% (v/v), with a pH of 3.8–4.0, and the type of alcohol was determined as ethanol using an ultraviolet spectrometer at the Organic Chemistry Laboratory of Jimma University (Table 1).

### 3.2. Body Weight of the Mice

The paired *t*-test revealed that the mean final body weight of Group I (control) mice (34.50 ± 2.35 g) significantly increased compared to their initial mean body weight (28.50 ± 2.88 g) (*p* < 0.05), There was an increase in the final body weight in Group 2, but this was not significant compared to the initial mean and as found in Group 1. In Group 3, however, the final body weight decreased but not significantly. In Group 4, the decrease was significant (Table 2).

### 3.3. Liver Tissue Histology

**Figure 1:**
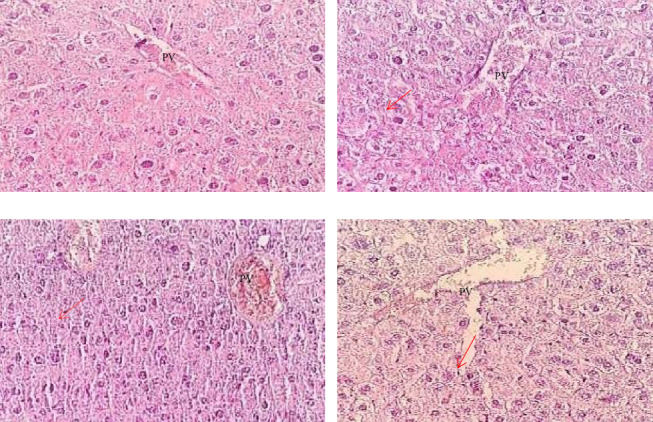
The photomicrographs of liver tissue sections from mice were stained with H&E, and all photomicrographs were taken at a magnification of 40X. (a) The control group consumed 1 mL of distilled water/kg, (b) 1 mL of 20% of Arak/kg, (c) 1 mL of 40% of Arak/kg, and (d) 1 mL of 40% of Arak/kg during the experimentation period. The arrow indicates a condition of small fat droplets and hepatocyte necrosis in the Arak-consumed groups. PV: portal vein.

In this study, the liver tissue of the control group has a normal histological structure, a normal central vein, and sinusoidal capillaries with no evidence of narrowing and no change in the hepatocyte's cytoplasm and nucleus, as shown in Figure 1(a). Under a simple microscope, there were inflammations and necrosis in the liver tissue of the Group II or (Figure 1(b)) Group III mice (Figure 1(c)) and Group IV (Figure 1(d)) mice, the degree of which varies depending on the concentration of ethanol in the Arak (Figure 1).

### 3.4. Liver Function Biomarkers

According to the study, there was a significant difference in serum AST and ALT levels between the control and experimental groups. There was a significant increment of serum ALT and AST in the experimental groups when compared to the control group. The serum AST level of Group III mice (169.83 ± 15.91 U/L) significantly increased compared to Group II mice (87.67 ± 38.85 U/L) (*p* < 0.05), and the serum AST level of Group IV mice (178.50 ± 12.42 U/L) was also increased compared to Group III mice but not significantly (*p* > 0.05); the serum ALT level of Group II mice (46.67 ± 21.50 U/L) was significantly greater than Group I mice (19.83 ± 11.92 U/L) (*p* < 0.05), and the serum ALT level of Group IV mice (71.00 ± 13.91U/L) was increased compared to Group III (61.33 ± 29.87) but not significantly (*p* > 0.05) (Table 3).

### 3.5. Kidney Tissue Histology

**Figure 2:**
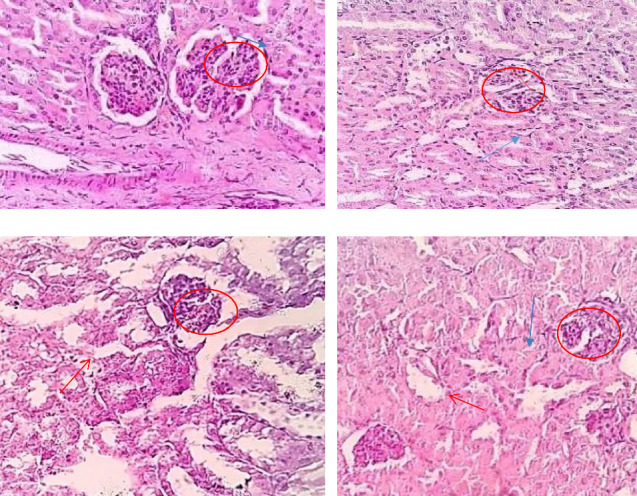
Photomicrographs of kidney sections stained with H&E were taken from mice at a magnification of 40X, with Group I (a) as the control with distilled water/kg, Group II (b) with 1 mL/kg of 20% Arak/kg, Group III (c) with 1 mL/kg of 40% Arak/kg, and Group IV (d) consumed with 40% Arak/kg during the experimentation period; the photomicrographs showed small fat droplets indicated by a red arrow and Bowman's capsule necrosis denoted by the blue arrow in alcohol-consumed groups, while the nonconsumed group showed a normal Bowman's capsule.

In this study, the kidney tissue of the control group (Figure 2(a)) has a normal histological appearance of glomeruli, renal tubules, and basement membrane as well. However, there were histopathological changes in the kidney tissues of the experimental groups. There was inflammation, swelling (Figure 2(c)), fat accumulation (obscure Bowman's space, Figure 2(b)), foamy appearances, and necrosis of the renal parenchyma as shown in Figure 2(d) in mice administered with Arak during the study period (Figure 2).

### 3.6. Kidney Function Biomarkers

The serum urea level of Group III (57.00 ± 16.95 mg/dL) and Group IV mice (70.17 ± 9.00 mg/dL) significantly increased compared to Group I mice serum urea level (16.00 ± 9.74 mg/dL) (*p* < 0.05), and the serum urea level of Group IV (40.83 ± 0.12 mg/dL) significantly increased compared to the serum urea level of Group I (control group) mice (39.59 ± 0.22 mg/dL) (*p* < 0.05), while the serum urea level of Group II (57.00 ± 16.95 mg/dL) and Group III (57.00 ± 16.95 mg/dL) mice increased but not significantly (*p* > 0.05); there was an insignificant increment in the serum creatinine level of experimental groups compared to the control group (*p* > 0.05), and among experimental groups, the serum creatinine level of Group III (1.00 mg/dL) and Group IV (1.00 mg/dL) mice increased compared to the serum creatinine level of Group II mice (0.83 ± 0.41 mg/dl) (*p* > 0.05), but the difference between all groups was insignificant (*p* > 0.05) (Table 4).

## 4. Discussions

Arak is the most commonly consumed traditionally prepared alcoholic drink in Ethiopia. However, the acute hepatorenal alteration induced by Arak is not well studied. Thus, this study aims to evaluate the hepatorenal alteration induced by traditionally prepared Arak in Swiss albino mice. The findings from this study indicated that the alcoholic contents of the collected Arak were 20%, 40%, and 45% (v/v), with a pH of 3.8–4.0 using an alcoholmeter. Several studies reveal that the ethanol content and pH of Arak vary depending on the distillation time, the raw materials used, and the skill of the person who produces it [34].

### 4.1. Body Weight of the Mice

The findings from this study showed that the final mean body weight of Group I significantly increased and Group II's mean body weight slightly decreased when compared to Group III mice. The reason behind this might be that factors including genetic, environmental, physiological, and behavioral influence body weight, and inappropriate alcohol intake is also one of the factors that lead to body weight change [35, 36]. Furthermore, the final mean body weight of 1 mL of 45%/kg consumed by mice was decreased with a mean difference of 1.45 ± 2.19 (*p* < 0.001) when compared to the initial body weight. The findings of this study contrast with a study performed in America by Wannamethee and his colleagues, which states that alcohol consumption increases the risk of obesity and weight gain [38]. The discrepancy may be due to study species variation, production process, and other microingredients found in the alcohol.

In this study, the body weight of the consumed groups slightly decreased as the dose of Arak provided was increased. The findings of this study are in contrast to a study performed in Poland by Alexandra et al. in 2019 and colleagues on the influence of alcohol consumption on body mass gain in growing adolescent Wistar rats [37]. The variation may be due to species variation between mice and Wistar rats, and the alcohol consumed may have biochemical differences (ingredients). However, the finding of this study is consistent with a study conducted in Croatia in 2017 by Tong Zhou et al. on the protective effects of lemon juice on alcohol-induced liver injury, which revealed that ethanol consumption reduces body weight in mice [38, 39].

### 4.2. Liver Tissue Histology

In this study, the liver tissue of the control group has a normal histological structure, a normal central vein, and sinusoidal capillaries with no evidence of narrowing and no change in the hepatocyte's cytoplasm and nucleus, as shown in Figure 2(a). Under a simple microscope, there were inflammations and necrosis in the liver tissue of the Group II or (Figure 2(b)) Group III mice (Figure 2(c)) and Group IV (Figure 2(d)) mice, the degree of which varies depending on the concentration of ethanol in the Arak. This study is comparable to a study that reported that ethanol affects protein turnover and induces oxidative stress [40, 41]. In addition to inflammation and necrosis, there was a shrinking of hepatocytes and fibrotic tissue observed in Groups III (Figure 2(c)) and IV (Figure 2(d)) mice. This finding is also comparable with the study because 1 mL/kg of 20% Arak for a long duration leads to the persistence of blood ethanol at high levels and severe lesion development [42]. This study revealed dose-dependent inflammation and necrosis of hepatocytes in mice administered with 1 mL of 20%/kg Arak (Figure 2(b)), 1 mL of 40%/kg Arak (Figure 2(c)), and 1 mL of 45%/kg Arak (Figure 2(d)). The findings of this study were consistent with studies performed in China in 2017 by Tong Zhou et al. on alcohol-induced liver injury and in Iraq in 2015 on alcohol-induced hepatic and renal histopathological change, which showed peripheral inflammation and necrosis when prepared slides were viewed under a simple microscope [39, 43].

### 4.3. Liver Function Biomarkers

The serum AST level of mice provided with 1 mL of 40%/kg Arak significantly increased compared to mice provided with 1 mL of 20%/kg Arak but less compared to mice provided with 1 mL of 45%/kg Arak. The serum ALT level of 1 mL of 20%/kg was less than 1 mL of 45%/kg consumed by mice and 1 mL of 40%/kg. The serum AST and ALT levels are directly related to tissue damage [18]. As the dose of Arak administered increased, the serum AST level increased in the experimental groups. These findings are supported by a previous study by Darius et al. in Minnesota on using the ratio of AST and ALT as a potential indicator for differentiating nonalcoholic steatohepatitis from alcoholic liver diseases [44, 45]. The elevation of these liver enzymes in the serum is a biochemical analysis used as an indicator of liver tissue damage [46–49].

### 4.4. Kidney Tissue Histology

In this study, the kidney tissue of the control group (Figure 2(a)) has a normal histological appearance of glomeruli, renal tubules, and basement membrane as well. However, there were histopathological changes in the kidney tissues such as inflammation, swelling (Figure 2(c)), fat accumulation (obscure Bowman's space, Figure 2(b)), foamy appearances, and necrosis of the renal parenchyma as shown in Figure 2(d) in mice administered with Arak. This might be due to the ethanol in the Arak, which leads to sodium and potassium excretion or electrolyte imbalance and causes swelling [50–52]. The degree of inflammation and necrosis was exacerbated as the dose increased. This finding is similar to a study performed in the USA and Nigeria on rats, which stated that alcohol alters hepatocytes by inducing inflammation and shrinking Bowman's space [21, 53]. The reason may be due to alcohol-induced oxidative stress of polyunsaturated fatty acids in the composition of renal lipids, which can cause a degeneration of the renal tissue/inflammation, necrosis, and acid-base balance abnormalities [47, 51, 54].

### 4.5. Kidney Function Biomarkers

This study found a dose-dependent increment in serum creatinine levels across experimental groups. These results are similar to previous studies on rats, which showed the effects of exposure to ethanol on kidney oxidative damage [55]. The kidney damage in mice may be due to the formation of free radicals during alcohol metabolism, leading to oxidative stress in the cells [22, 56].

The results of this study revealed that serum creatinine levels rose with increasing doses of consumed Arak, without any accompanying histological changes. The reason might be that creatinine is the end product of the metabolism of creatinine phosphate, a byproduct of skeletal muscle metabolism, excreted via the kidney primarily by glomerular filtration [57, 58]. Functional acute kidney injury (not related to structural alteration) is related to muscle cell degradation, which has a protein content and is bypassed by the kidney as a byproduct of protein metabolism [59].

## 5. Conclusions

Arak had a significant dose-dependent effect on decreasing body weight, increasing serum levels of liver enzymes (AST and ALT), and elevating serum levels of BUN and creatinine in Swiss albino mice. The effect of the Arak consumption was approved through histopathological evaluations. Arak consumption harms liver and kidney tissues by causing inflammation, fat accumulation, and necrosis, with these effects intensified by elevated ethanol levels, as observed in Swiss albino mice. This study's findings suggest that Arak may have dose-dependent toxic effects on the liver and kidneys. Further studies are needed to evaluate its impact on human health.

### 5.1. Limitations of the Study

This study does not identify other biochemical components that could affect the results. Furthermore, this study lacks immunohistochemistry (IHC) or molecular endpoints, which could strengthen findings in future studies. Stratified analysis between the sexes and groups was not considered due to resource constraints and small sample size, and it does not identify the LD_50_ and LD_100_ of traditionally prepared Arak in the community.

## Figures and Tables

**Table 1:** Experimental evaluation of dose-dependent hepatorenal toxicity of traditionally prepared Arak in Swiss albino mice, and concentration (v/v) of randomly prepared Arak for the study.

Arak	Concentration of ethanol (v/v) (%)
Arak produced	40
Arak collected 1	20
Arak collected 2	45

**Table 2:** Experimental evaluation of dose-dependent hepatorenal toxicity of traditionally prepared Arak on the mean body weight in Swiss albino mice.

Groups	Initial mean W (g)	Final mean W (g)	Mean ± SD
I	28.50 ± 2.88	34.5 ± 2.35	4.17 ± 0.93^∗^
II	27.83 ± 2.48	30.17 ± 2.86	1.81 ± 1.91^∗∗^
III	29.00 ± 4.15	27.17 ± 3.60	0.52 ± 1.26^∗∗^
IV	29.67 ± 1.63	28.00 ± 2.00	1.45 ± 2.19^∗^

*Note:* The results are expressed as mean ± standard deviation.

^∗^ *p* values (*p* < 0.05) were statistically significant between the initial and final mean body weight.

^∗∗^ *p* values (*p* > 0.05) were not statistically significant.

**Table 3:** Experimental evaluation of dose-dependent hepatorenal toxicity of traditionally prepared Arak on some liver function parameters in Swiss albino mice.

Groups	Biochemical analysis of liver function	
	AST (U/L)	ALT (U/L)
Group I	43.17 ± 10.60^∗^	19.83 ± 11.92^∗^
Group II	87.67 ± 38.85^∗^	46.67 ± 21.50^∗^
Group III	169.83 ± 15.91^∗∗^	61.33 ± 29.87^∗∗^
Group IV	178.50 ± 12.42^∗∗^	71.00 ± 13.91^∗∗^

*Note:* The results are expressed as mean ± standard deviation.

^∗^Asterisks within the same column are statistically significant (*p* < 0.05).

^∗∗^Values within the same column were not significant (*p* > 0.05).

**Table 4:** Experimental evaluation of dose-dependent hepatorenal toxicity of traditionally prepared Arak on some renal function parameters in Swiss albino mice.

Groups	Biochemical analysis of kidney damage	
	BUN (mg/dL)	Creatinine (mg/dL)
Group I	16.00 ± 9.74^∗^	0.67 ± 0.52^∗^
Group II	40.83 ± 17.87^∗∗^	0.83 ± 0.41^∗∗^
Group III	57.00 ± 16.95^∗∗^	1.00^∗∗^
Group IV	70.17 ± 9.00^∗^	1.00^∗∗^

*Note:* The results are expressed as mean ± standard deviation.

^∗^Asterisks within the same column are statistically significant (*p* < 0.05).

^∗∗^Asterisks in the same column show no significance (*p* > 0.05).

## Data Availability

The data that support the findings of this study are available from the corresponding author upon reasonable request.

## Nomenclature

ALT: Alanine aminotransferase
APC: Alcohol consumption per capital
AST: Aspartate aminotransferase
BUN: Bilirubin, urea, and nitrogen
DPX: Dibutyl phthalate polystyrenes xylene
IHC: Immunohistochemistry
IRB: Institutional review board
JIT: Jimma Institute of Technology
JUCAVM: Jimma University College of Agriculture and Veterinary Medicine
NBF: Neutral buffered formalin
PLC: Private limited company
ROS: Reactive oxygen species
UDAW: Universal Declaration of Animal Welfare
USA: United States of America
UV: Ultraviolet
